# New Pulsed Cold Neutron Beam Line for Fundamental Nuclear Physics at LANSCE

**DOI:** 10.6028/jres.110.014

**Published:** 2005-06-01

**Authors:** P.-N. Seo, J. D. Bowman, M. Gericke, R. C. Gillis, G. L. Greene, M. B. Leuschner, J. Long, R. Mahurin, G. S. Mitchell, S. I. Penttila, G. Peralta, E. I. Sharapov, W. S. Wilburn

**Affiliations:** Los Alamos National Laboratory, Los Alamos, NM 87545, USA; Los Alamos National Laboratory, Los Alamos, NM 87545, USA; Indiana University, Bloomington, IN 47405, USA; University of Manitoba, Winnipeg, MB R3T2N2, Canada; Oak Ridge National Laboratory, Oak Ridge, TN 37831, USA; University of Tennessee, Knoxville, TN 37996, USA; Indiana University, Bloomington, IN 47405, USA; Los Alamos National Laboratory, Los Alamos, NM 87545, USA; University of Tennessee, Knoxville, TN 37996, USA; Los Alamos National Laboratory, Los Alamos, NM 87545, USA; Joint Institute for Nuclear Research, Dubna, Russia; Los Alamos National Laboratory, Los Alamos, NM 87545, USA

**Keywords:** hydrogen moderator, moderator brightness, neutron capture, neutron guide, polarized neutrons, spallation neutron source

## Abstract

The NPDGamma collaboration has completed the construction of a pulsed cold neutron beam line on flight path12 at the Los Alamos Neutron Science Center (LANSCE). We describe the new beam line and characteristics of the beam. We report results of the moderator brightness and the guide performance measurements. FP12 has the highest pulsed cold neutron intensity for nuclear physics in the world.

## 1. Introduction

The NPDGamma collaboration has constructed and commissioned a pulsed cold neutron beam line, flight path 12 (FP12), at LANSCE. This beam line is designated for basic nuclear physics research. It consists of a super-mirror (SM) neutron guide, shutter, and two frame-definition choppers. The pulsed nature of the neutron beam with a narrow proton pulse provides accurate neutron energy information through a time-of-flight (TOF) measurement and the possibility for absolute polarimetry to control systematic uncertainties in precision experiments. The first experiment will be the NPDGamma, $\vec{n}+p\to d+\gamma$, to study the hadronic weak interaction between nucleons [1]. NPDGamma will determine the weak pion-coupling constant, *f*_π_, by measuring a very small parity-violating directional gamma-ray asymmetry, *A_γ_*, in the reaction where polarized cold neutrons are captured on a para-hydrogen target. The predicted value of *A_γ_* is 5 × 10^−8^, which the experiment aims to determine with 10 % precision [2,3]. The final statistical sensitivity on *A_γ_* will depend upon the cold neutron flux on the hydrogen target.

## 2. The FP12 Moderator

From the LANSCE linear accelerator 800 MeV H^−^ beam pulses are injected into the Proton Storage Ring (PSR). As a part of the injection process, the H^−^ particles are stripped to H^+^. In the PSR the protons are accumulated and compressed into pulses with a roughly triangular shape, 250 ns wide at the base. The proton pulses are then extracted to a tungsten neutron production target at the rate of 20 Hz and with average current of ≈100 µA [4]. Energetic neutrons from the spallation process are moderated with water or cold-hydrogen moderators. Two out of six moderators at LANSCE are hydrogen moderators operated with supercritical hydrogen gas. The FP12 and FP13 neutron guides view a unique partially-decoupled cold hydrogen moderator in a backscattering and flux-trapped geometry [5]. The 12 cm by 12 cm surface area of the moderator is perpendicular to FP12. The brightness of the moderator was estimated by MCNP modeling [5,6]. Since the sensitivity of the NPDGamma experiment is determined by the neutron statistics, we decided to measure the brightness of the FP12 moderator. The measurement was done with a novel two-pinhole collimator system and a ^6^Li-loaded scintillation neutron detector. The measurement and result are described in detail in Ref. [7]. The brightness has a maximum of 1.3 × 10^8^ n/s/cm^2^/sr/meV/µA with 7 % uncertainty at 3 meV neutron energy.

The neutron pulse from the moderator has two decay time constants. During the moderation process, neutrons in a thermal energy range experience more than one collision with hydrogen molecules in the moderator and with particles in the Be-reflector that surrounds the moderator. The shorter time constant, ≈250 µs, is related to the hydrogen moderation and the longer time constant, ≈600 µs, is caused by the Be-reflector. When the neutron energy decreases, more neutrons have the longer time constant [8].

## 3. The FP12 Neutron Guide

**Fig. 1:**
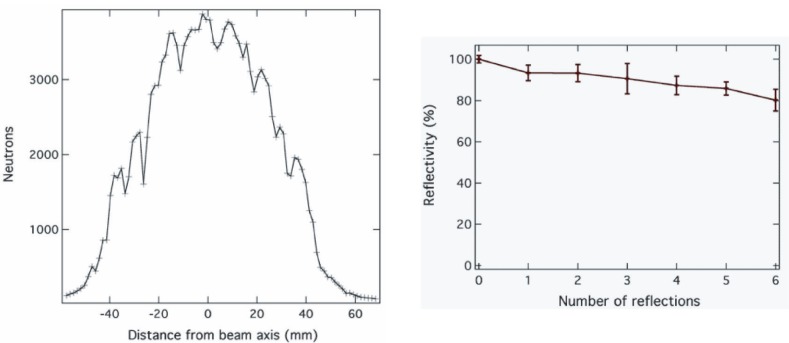
The left plot is the number of 3-meV (0.56 nm) neutrons within a 2.4-ms gate width for 10s. The right plot shows the measured reflectivity of the installed 21-m long neutron guide as a function of a number of reflections.

Neutrons from the moderator enter the 21 m long straight *m* = *θ*_c_/*θ_c_* (^nat^Ni) = 3 SM neutron guide [9], where *θ*_c_ is the critical angle of reflection. The inner cross section of the guide is 9.5 cm × 9.5 cm. The transmission of the guide is based on the total reflection of neutrons on the inner walls of the guide. Reflectivity of every 50 cm long guide element was measured with 0.427 nm neutrons by the manufacturer and it is better than 85 % for a glancing angle *m* = 3 [9]. We measured the reflectivity of the installed 21 m long guide with the two-pinhole collimator system [7]. Figure 1 (left) shows 3 meV (0.53 nm) neutrons when the detector-downstream pinhole system was moved up-down respect to the beam axis. When this system is moved farther from the beam axis, neutrons have to have larger glancing angles in order to enter the detector: they go through a number of reflections in the guide. The maximum number of reflections with this collimation for a 3 meV neutron is six. Each peak in the plot represents a different number of reflections. From the results of the vertical (up-down) and horizontal (left-right) scans, an average reflectivity per neutron reflection was extracted as shown in Fig. 1 (right). Our results show that the two-pinhole system is a simple and sensitive method to measure a moderator brightness, to study reflectivity, and alignment of a neutron guide.

**Fig. 2:**
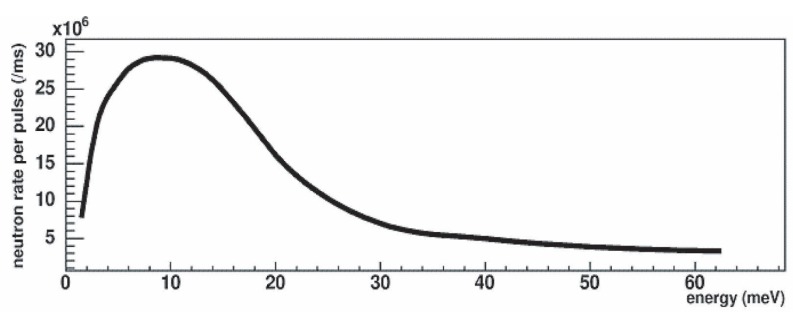
Monte Carlo calculated neutron flux out of the real FP12 guide as a function of neutron energy (top) and of time-of-flight (bottom). The fast neutron part of the spectrum was not included in the calculation.

Based on the result of the moderator brightness measurement, which was done after installing the first part of the full guide, we calculated by Monte Carlo the neutron flux out of the full length of the guide. The flux is plotted in Fig. 2 as a function of neutron energy (top) and TOF (bottom) for an average proton current of 95 µA.

## 4. Frame-Definition Choppers

**Fig. 3:**
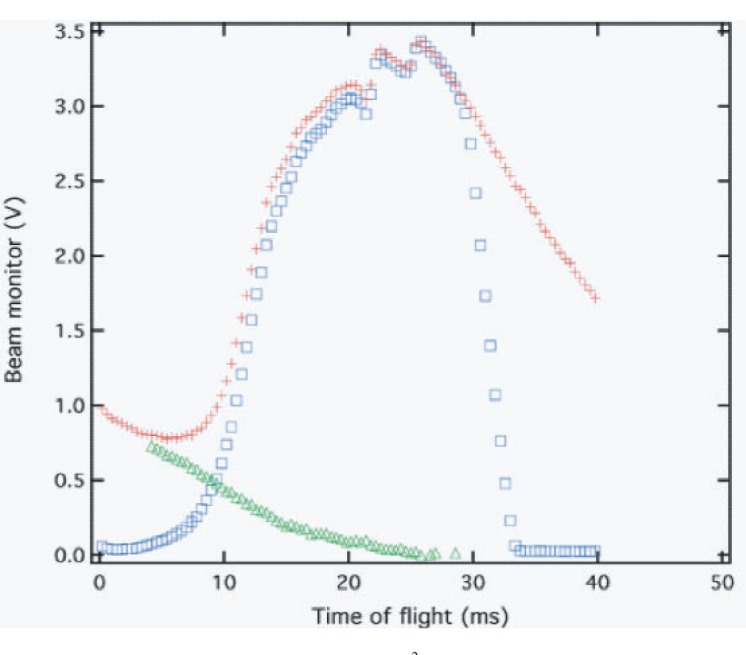
Time-of-flight spectra measured by a ^3^He ion chamber at to the end of the guide in downstream. The spectrum (+) is for neutrons with the chopper-off and the spectrum (□) for neutrons with the chopper-on and phased to *T*_0_, respectively. The spectrum (△) shows the contribution of slow neutrons from the previous frame when the chopper is not running.

The beam line has two rotating (1200 rpm) frame-definition choppers located at 9.38 m from the surface of the moderator. The choppers are used to define the TOF range of interest and to prevent low-energy neutrons from the previous frame from entering the new frame. Since the flight path is about 21 m and the full TOF frame is 50 ms, the slowest neutrons that reach the experiment at the end of each frame have 1 meV energy. To block undesired neutrons, the chopper aluminum plates were coated with Gd_2_O_3_. The thickness of the absorber layer was determined to be black for 30 meV neutrons. The diameter of the chopper plates is 1024 mm. Each chopper plate has a 109° opening for beams. It takes 1.8 ms for the edge of the beam aperture to cross the full guide. At 21 m from the neutron source the aperture opening or closing takes 4.0 ms as seen from Fig. 3.

The performance of one of the frame definition choppers is shown in Fig. 3, where time-of-flight spectra obtained with a ^3^He beam monitor mounted on the end of the guide. The spectrum (Δ) shows the contribution of slow neutrons from the previous frame when the chopper is not running. A spectrum (+) was measured with the chopper-off and thus, neutrons from the previous frame were detected. A spectrum () was taken with the chopper running and was phased to *T*_0_. The beam aperture starts to open at 0 ms, is fully open at 4 ms, and starts to close at 30 ms. Note that the full length of the time-of-flight frame is 50 ms. The last 10 ms is used by the data acquisition system to transfer data. The fast neutron part of the spectrum was not detected because of the small n-^3^He absorption cross section and the small ^3^He thickness of the monitor. With two independent choppers any length of the time-of-flight period shorter than 26 ms can be selected. The choppers are tightly phased to the facility master-timing-reference which in turn is referenced to the power grid. The same timing is used for the proton extraction from the PSR. The chopper feed-back loop keeps the chopper phased to *T*_0_ in 50 µs. The chopper-closed part of the time-of-flight spectrum is used by the NPDGamma to study detector pedestals and beta decay from neutron activation.
